# Competence over case count: rethinking paediatric orthopaedic training

**DOI:** 10.1308/rcsann.2025.0105

**Published:** 2025-12-15

**Authors:** AT Schade, A Pilarski, D Waugh, D Eastwood

**Affiliations:** ^1^ University Hospitals Coventry and Warwickshire NHS Trust, UK; ^2^ Malawi-Liverpool-Wellcome Trust, Blantyre, Malawi; ^3^ NHS Greater Glasgow and Clyde, UK; ^4^ Royal National Orthopaedic Hospital NHS Trust, UK

The recent paper on paediatric operative exposure among United Kingdom (UK) orthopaedic trainees provides an important reflection on how our training systems must evolve alongside the changing practice of paediatric orthopaedics, and prompt a broader discussion about what constitutes meaningful paediatric orthopaedic training in the modern era.^1^

The authors highlight that trainees are achieving Certificate of Completion of Training (CCT) with limited exposure to key paediatric orthopaedic procedures listed in the Joint Committee on Surgical Training (JCST) curriculum.^2^ These operations are becoming increasingly uncommon in UK practice, driven by lower national birth rates and centralisation of paediatric services to tertiary centres.^3,4^ A limitation of the study is the lack of granularity in the e-logbook data. The authors did not report how often trainees assisted in these key trauma procedures or how many workplace-based assessments were completed for each condition. Moreover, although the authors suggest that general orthopaedic surgeons may be less suited to managing slipped upper femoral epiphysis (SUFE) without specialist input, they present no data on where procedures were performed or under whose supervision. Many district hospitals do not routinely operate on children, yet certain procedures, such as septic hip washout and in situ pinning of SUFE, are time critical and must remain deliverable in district general hospitals with appropriate paediatric anaesthetic support, rather than being limited to tertiary centres.^5^ The physiology and pathology of sepsis in a 6-month-old child differ markedly from those in an adolescent, and training as well as management algorithms must account for these distinctions. The growing adoption of arthrocentesis and arthroscopy, which challenge the traditional requirement to ‘open’ a septic joint, has consequently limited trainees’ exposure to arthrotomy when definitive treatment is indicated.^6,7^ How paediatric experience is best captured remains uncertain, whether through the e-logbook, workplace-based assessments, or more qualitative and observational study methods. Addressing the mismatch between training exposure and JCST requirements will require better evidence to inform training, alongside modernised assessment methods, greater use of simulation and regional rotations, and national collaboration to ensure equitable, competency-based learning.^8^

The majority of paediatric trauma procedures recorded in trainee logbooks are manipulations under anaesthetic and cast applications, reflecting a positive shift in practice as we celebrate the growing body of evidence supporting non-operative management.^9–11^ Non-operative management skills are often undervalued in traditional training structures and should be explicitly recognised within the e-logbook and workplace-based assessments and supplemented through simulation and dedicated courses. Recognising and standardising a broader definition of paediatric trauma competence will ensure training remains aligned with modern practice.

The reduction in operative exposure extends beyond paediatric orthopaedics and reflects a wider trend across surgical training. Although the authors note the impact of the COVID-19 pandemic, there is growing evidence that many other factors contribute to this decline. Trainees face increasing service demands, with heavier on-call workloads and fewer operative opportunities.^12^ The expanding administrative requirements associated with electronic patient records, audits, national registries and recording of evidence in portfolios have further eroded time available for hands-on training.^13^ Training programmes must evolve to increase both the number and value of training opportunities available within the current time frame. Globally, orthopaedic training is completed within shorter time frames, and the UK priority should be to optimise the efficiency and educational value of existing posts, ensuring trainees achieve competency-based outcomes within the current structure.^14^

In summary, the study highlights a growing mismatch between curriculum expectations and real-world paediatric experience. Training must move beyond counting cases to focus on competence, incorporating both operative and non-operative skills. By modernising assessment methods, optimising clinical opportunities, and leveraging simulation, regional rotations and digital tools, we can ensure trainees achieve the competencies required for CCT within current training structures. Ultimately, training in paediatric orthopaedics must evolve to reflect contemporary practice while maintaining high standards of care for children.

## Competing interests

The author/s declare no competing interests.

## Funding

The author/s received no financial support for the research, authorship, and/or publication of this article.

## Author contributions

**AT Schade**: Conceptualisation, Supervision, Writing – original draft, Writing – review & editing. **D Waugh**: Writing – review & editing. **A Pilarski**: Writing – original draft, Writing – review & editing. **D Eastwood**: Supervision, Writing – review & editing.

## Artificial Intelligence

The author/s declare that no AI was used to conduct the study or prepare the manuscript.
